# Mineral Phase-Resolved Quantification in LA-ICP-MS Imaging

**DOI:** 10.1021/acs.analchem.5c05398

**Published:** 2025-12-17

**Authors:** Barbara Umfahrer, Jakub Buday, Pavel Pořízka, Jozef Kaiser, Paolo S. Garofalo, Detlef Günther

**Affiliations:** † Laboratory of Inorganic Chemistry, Department of Chemistry and Applied Biosciences, ETH Zurich, Vladimir-Prelog-Weg 1, Zurich 8093, Switzerland; ‡ 232848 Central European Institute of Technology (CEITEC) Brno University of Technology, Purkyňova 123, Brno 612 00, Czech Republic; § Dipartimento di Scienze Biologiche, 9296 Università degli Studi di Bologna, Geologiche ed Ambientali, Via Zamboni 67, Bologna 40126, Italy

## Abstract

Laser Ablation-Inductively Coupled Plasma-Mass Spectrometry (LA-ICP-MS), particularly in its time-of-flight (TOF) configuration, enables rapid, high-resolution elemental imaging across complex geological materials, offering spatial and chemical insights at the micrometer scale. However, quantitative accuracy is often limited in fine-grained or mineralogically heterogeneous matrices due to the failure of global normalization strategies, such as 100 wt % oxide assumptions, to account for mixed-phase compositions. Here, we present a workflow that leverages Uniform Manifold Approximation and Projection (UMAP) for unsupervised dimensionality reduction and *k*-means clustering to segment mineralogical phases directly from per-pixel elemental concentration maps. Cluster compositions are matched to known minerals based on stoichiometric similarity, enabling pixel-wise, phase-specific normalization (e.g., oxides vs carbonates). Validated with dawsonite-bearing sandstones from Mt. Amiata, Italy, this approach significantly reduces quantification errors, correcting systematic over- or underestimations of up to 60%. The method also enables a consistent, phase-resolved geochemical comparison across depth profiles. This study establishes UMAP not only as an exploratory tool but also as a practical guideline for accurate and interpretable quantification in multielemental imaging.

## Graphical abstract

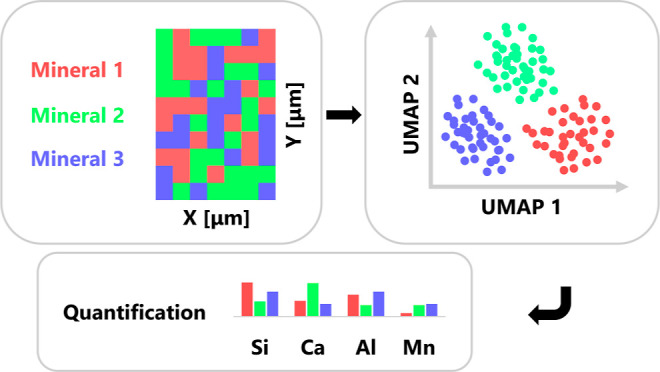

## Introduction

Since its introduction in 1985,^1^ Laser Ablation-Inductively Coupled Plasma-Mass Spectrometry (LA-ICP-MS) has matured into a routine technique for both qualitative and quantitative elemental analysis, finding use in diverse fields such as geology,^2–4^ biology,^5,6^ materials science,^7^ and cultural heritage.^8,9^ Especially in comparison to liquid analysis techniques, the method excels through minimal sample preparation, negligible sample destruction, and low limits of detection, which enable trace element analysis. However, besides the availability of standard reference materials, a longstanding challenge of quantitative LA-ICP-MS is the need for a suitable internal standard to account for variations in ablation rate and transport efficiency, as well as instrumental drift.^10^ Developed for applications in geological matrices, this requirement was elegantly circumvented by Liu et al.^11^ through 100 wt % normalization, which assumes that all detected elements form oxides summing up to 100 wt %. This approach was conceptually inspired by the summed-spectrum normalization calibration procedure introduced by Leach and Hieftje in 2000, which emphasized the use of total signal intensities for robust internal standardization.^12^ The 100 wt % normalization strategy was subsequently extended to the bulk analysis of carbonates.^13^

Concurrently, the advent of time-of-flight (TOF) mass analyzers and the development of fast-washout laser ablation cells^14–16^ have transformed LA-ICP-MS into a powerful imaging tool, capable of generating spatial maps across a variety of sample matrices at high data acquisition rates.^17–19^ Furthermore, in comparison to, e.g., X-ray fluorescence spectroscopy (XRF) or Scanning Electron Microscopy with Energy-Dispersive X-ray Spectroscopy (SEM-EDS), LA-ICP-MS offers a high depth resolution of approximately 0.2 μm on glass, enabling precise measurement of fine-scale structures not only laterally but also in depth.^18^ However, as the volume and complexity of these elemental images have grown according to the progressive advancement of the method itself, now consisting of gigabytes of data produced within minutes, manual data interpretation has become increasingly impractical. To uncover meaningful details and patterns, researchers have applied multivariate methods such as Principal Component Analysis (PCA) and k-means clustering to reduce dimensionality and delineate distinct regions within the images.^20–23^ The nonlinear dimensionality reduction technique Uniform Manifold Approximation and Projection (UMAP),^24^ which supports data interpretation with its capability to capture and separate subtle local structures, has first been employed to LA-ICP-MS bulk analysis data in 2024, where it was used to classify pyrite samples and discriminate between Pb and Zn deposit types.^25,26^ In combination with elemental imaging, UMAP has recently been applied to biological tissue data sets as an exploratory analysis tool,^27^ further enhancing the ability to visualize and interpret complex elemental distributions.

Despite these advances, no existing approach combines the unsupervised segmentation of mineral phases by UMAP with phase-specific normalization. In this study, we introduce a UMAP-driven workflow that (1) automatically segments distinct mineralogical phases within fine-grained geological matrices and (2) applies phase-specific 100 wt % normalization (e.g., oxide^11^ or carbonate^13^), thereby improving quantification accuracy and enabling phase-specific quantitative interpretation of mineral distributions in complex matrices.

## Materials and Methods

### Samples

The geological matrix is a fine-grained sedimentary rock (sandstone) forming the caprocks of the Mt. Amiata geothermal system (Sillano-Santa Fiora Formation of S. Tuscany, Italy).^28,29^ It is a carbonate-bearing sandstone collected in a locality called Poggio del Gatto (Coordinates: N 42° 50.5351; E 11° 41.0451’; Elevation: 650 m) close to the city of Piancastagnaio, which hosts peculiar geothermal mineralizations including dawsonite (NaAlCO_3_(OH)_2_). This carbonate mineral was described in geological environments dominated by volcanic rocks^30^ and is under investigation for its ability to sequester and store CO_2_ as an option to reduce global greenhouse gas emissions.^31^ To probe this process, we characterized this fine-grained, multiphase sandstone. The initial geological objective for which this method was developed was to resolve and quantify the complex compositional variability of the distinct minerals forming the dawsonite-bearing sedimentary rocks of Mt. Amiata.

The samples studied consist of four thin sections of the Poggio del Gatto sandstone, which were prepared from four adjacent blocks (herein named “Layer 1” to “Layer 4”) of a larger sample of about 3 kg (see Figure S1). Being located very close to each other, these layers allow determining mesoscale changes of mineral and chemical compositions of the rock and are fundamental to quantify fluid–rock interactions.^32^ X-ray diffraction (XRD) measurements of the four blocks identified the presence of quartz, calcite, kaolinite, white mica, and plagioclase. The ideal stoichiometries of the minerals identified are listed in the Supporting Information under Table S1. Thin section photomicrographs were acquired on a polarizing microscope (Olympus BX51, Tokyo, Japan) in transmitted light as an aid in mineral identification.

XRD analyses were conducted at the Analitica Lab of San Lazzaro di Savena (Bologna, Italy) by using a GNR APD2000PRO diffractometer with Cu Kα radiation equipped with a secondary graphite monochromator. The four blocks were powdered and prepared by filling a side-entry aluminum holder to obtain a quasi-random orientation of each sample on a glass plate. Analyses were conducted after drying the plates at ca. 40 °C to eliminate adsorbed water on the sheet silicates. Mineral identification was obtained processing the XRD patterns with the Profex software^33^ and comparing the patterns with reference data supplied by the Crystallography Open Database (COD) (https://www.crystallography.net/cod/).

### LA-ICP-TOFMS Imaging

LA-ICP-TOFMS analyses were performed using a 193 nm ArF excimer laser (GeoLas C, Lambda Physik, Göttingen, Germany) coupled to an ICP-TOFMS instrument (icpTOF 2R, TOFWERK AG, Thun, Switzerland). Ablation was conducted in the modified parallel flow ablation cell (MPFAC)^16,34^ under a He atmosphere (99.999%, Linde Gas Schweiz AG, Dagmersellen, Switzerland) with Ar (99.996%, Linde Gas Schweiz AG) used as the carrier gas. A piezoelectrically driven xyz translational stage (1 nm resolution, SmarAct GmbH, Oldenburg, Germany) enabled precise sample positioning. Laser, stage movement, and ICP-TOFMS data acquisition were synchronized using a custom-built imaging control system.^19^ The system was optimized each day based on maximum sensitivity while ensuring a ^238^U^+^/^232^Th^+^ intensity ratio of 1.0 to 1.1 on NIST SRM 610 and a ^232^Th^16^O^+^/^232^Th^+^ rate of below 0.5%.

Samples were ablated in hole-drilling mode with 10 shots per pixel before advancing to the next position. Consecutive ablation spots were aligned edge to edge. Two areas measuring 500 μm × 500 μm each were analyzed and combined afterward. The external standard NIST SRM 610 was measured before, between, and after the sample analyses to monitor and correct for potential instrumental drift. Average gas blank signal intensities, measured over 2 min, were subtracted from all measurements. The instrument parameters are summarized in Table 1.

**Table 1:** LA-ICP-TOFMS Instrument Parameters for the Imaging of the Thin Sections

laser		ICP-TOFMS	
fluence [J cm^–2^]	25	Ar [L min^–1^]	0.78–0.80
spot size [μm]	5	He [L min^–1^]	0.91–0.98
shots per pixel [ ]	10	sampling depth [mm]	3
repetition rate [Hz]	100	H_2_ [mL min^–1^]	2

### Data Processing and Statistical Analysis

#### Preprocessing and Oxide Normalization

Raw data were first processed in TOFWARE (v2.5.11, TOFWERK AG, Thun, Switzerland) to sum spectra and define integration boundaries. Peak intensities from both, samples and the reference material, were then processed using the Python code developed by Neff et al. in 2020.^19^ Elemental concentration values for the reference material NIST SRM 610 were obtained from the GeoReM database.^35^ Elemental maps were quantified per pixel using 100 wt % mass normalization,^11^ in the first instance and as a starting approach assuming all components to be oxides.

#### UMAP Embedding and *k*-Means Clustering

To refine the oxide-only assumption and better reflect mineralogical variability, mineral phases were identified on the quantified and all-oxide normalized elemental maps using *k*-means clustering on a UMAP embedding. The subsequent statistical analysis was conducted using Python 3.11.8 within the Spyder environment and open-source packages.

First, from every elemental map, binary masks were generated using Otsu thresholding^36^ and connected regions were labeled to extract the per-region mean and standard deviation across all elements. This ensured that only geochemically relevant areas, treated as “regions” corresponding to individual grains in the matrix, were included in subsequent processing. From the regional mean compositions, additional features were derived to enhance the discriminatory power of the data. These included elemental ratios, log_1+*x*_, square-root, and z-score transformations. The combined feature set was then filtered by retaining features with the highest variance and lowest interfeature correlation to reduce noise and redundancy. These selected features were uniformly scaled to the [0,1] range and embedded into a lower dimensional feature space using UMAP. Afterward, *k*-means was applied to the UMAP low dimensional feature space vectors, yielding region clusters. In contrast to the five minerals identified by XRD, overclustering with *k* = 10 was intentionally employed to capture subtle compositional variations, particularly within solid-solution series such as plagioclase.

UMAP hyperparameters were optimized via Bayesian optimization using the Optuna framework. The objective function maximized the silhouette score of the *k*-means clusters, penalized by the logarithm of the mean relative standard deviation (RSD) of major elements within each cluster. In this way, the objective is to balance cluster cohesion with compositional consistency within the identified clusters. Optimization was typically converged within 20–30 iterations (see Figure S2 in the Supporting Information). The UMAP parameter ranges used during optimization are summarized in Table 2. The optimized UMAP embedding and clustering results were spatially projected back onto the original image coordinates, generating computationally derived phase maps that were subsequently validated against the optical microscopy of the sample.

**Table 2:** Range of Hyperparameters during the Optimization Procedure of the UMAP Model

tuning range of hyperparameters	
n_components	2, 3
n_neighbors	5–100
min_dist	0.00001–0.1
spread	≤3.0
densmap	on, off
metric	cosine, correlation

#### Mineral Matching and Phase-Specific Renormalization

Once clusters were defined, their average compositions were converted from wt % to molar fractions to compute per-cluster stoichiometries. These clusters’ stoichiometries were compared to the stoichiometries of the minerals identified with XRD (ideal mineral compositions). The best-matching mineral was determined based on the minimum cosine distance between normalized stoichiometries. To assess the robustness of cosine distance matching of the mineral phases, the Relative match confidence is introduced to determine how distinctly the best match outperforms the second-best match. Specifically, for every UMAP cluster, the Relative match confidence is defined as

1 $$Relative\;match\;confidence=\frac{d_{\sec ond}-d_{best}}{d_{\sec ond}}$$

where *d*_best_ and *d*_second_ are the cosine distances to the closest and second-closest minerals, respectively (0 = tie; 1 = unambiguous match). Once the minerals are identified, overclustering is reversed by merging clusters corresponding to the same mineral phase, including the solid solutions of plagioclase. The carbonate phases are pixel-wise renormalized to 100 wt % carbonates.

### LIBS Imaging

The LIBS analyses were performed on a Firefly system (Lightigo, Czech Republic). The laser (1064 nm, 5 mJ, pulse duration ∼10 ns, diameter 6 mm, operating at 50 Hz) was focused on the sample with a singlet lens (focal length 50 mm) to a 30 μm spot. The spatial resolution of the acquired elemental maps, with a size of about 16 mm × 26 mm, was 20 μm. Plasma radiation is collected by a multifiber configuration with a collimator and an optical fiber (400 μm core diameter), which delivered the light to several Czerny–Turner spectrometers (resolution 0.25–0.4 nm). The spectral range of the spectrometers is 180–850 nm. The radiation is collected 1.5 μs after ablation, with a gate width of 15 μs.

Data processing was done with Python 3.11.8 in the Spyder environment and included background correction and per-pixel z-score normalization.

## Results and Discussion

### LIBS and XRD Measurements

To assess whether carbonate-based normalization^13^ is warranted, first, large-scale LIBS elemental intensity maps of the samples across about a third of the thin sections were acquired. Figure 1 reveals the results for “Layer 2” of the Poggio del Gatto sandstone for selected elements. A complete mapping of all four thin sections can be found in Figures S3 and S6 in the Supporting Information. The maps reveal that C is strongly enriched in fracture veins as well as in finer-grained domains within the matrix and correlates with Ca, indicating carbonate minerals rather than oxides. Bulk XRD analysis identifies calcite alongside quartz, plagioclase, white mica, and kaolinite. These complementary observations confirm calcite as a major phase and thus justify the need for performing the 100 wt % normalization on a carbonate basis for pixels representing calcite.

**Figure 1:**
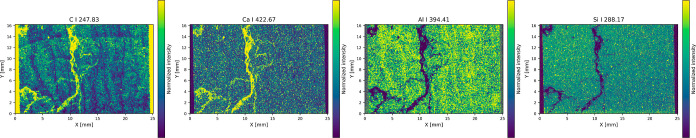
LIBS elemental intensity maps of the data set from “Layer 2” of the Poggio del Gatto sandstone mounted on a microscope slide, depicting calcite veins and fine-grained structures within the matrix, underlining the need for carbonate-based normalization for specific pixels. Emission lines for evaluation are shown in the corresponding subfigure title. The epoxy used to fix the thin section on the slide is visible along the margin.

### Phase Segmentation with UMAP

Figure 2 shows the optimized UMAP embedding alongside the corresponding computationally generated phase map. For illustration, we focus on “Layer 2” of the Poggio del Gatto sandstone; results for the remaining three thin sections (“Layer 1”, “Layer 3”, and “Layer 4”) are provided in the Supporting Information in Figures S7–S9. In this initial segmentation, cluster identities correspond to the selected number of clusters *k* in the *k*-means algorithm. The visualization shows that clusters identified in feature space translate into spatially coherent regions, though at this stage they are not interpretable as geological phases. As noted previously, we deliberately overclustered relative to the XRD-identified mineral assemblage. The relatively minor plagioclase (whose abundance in the rock was evaluated at 12–14 wt % by XRD) forms extensive solid solutions (named labradorite, bytownite, oligoclase, anorthite, albite) and is therefore difficult to subdivide univocally as one cluster.

**Figure 2:**
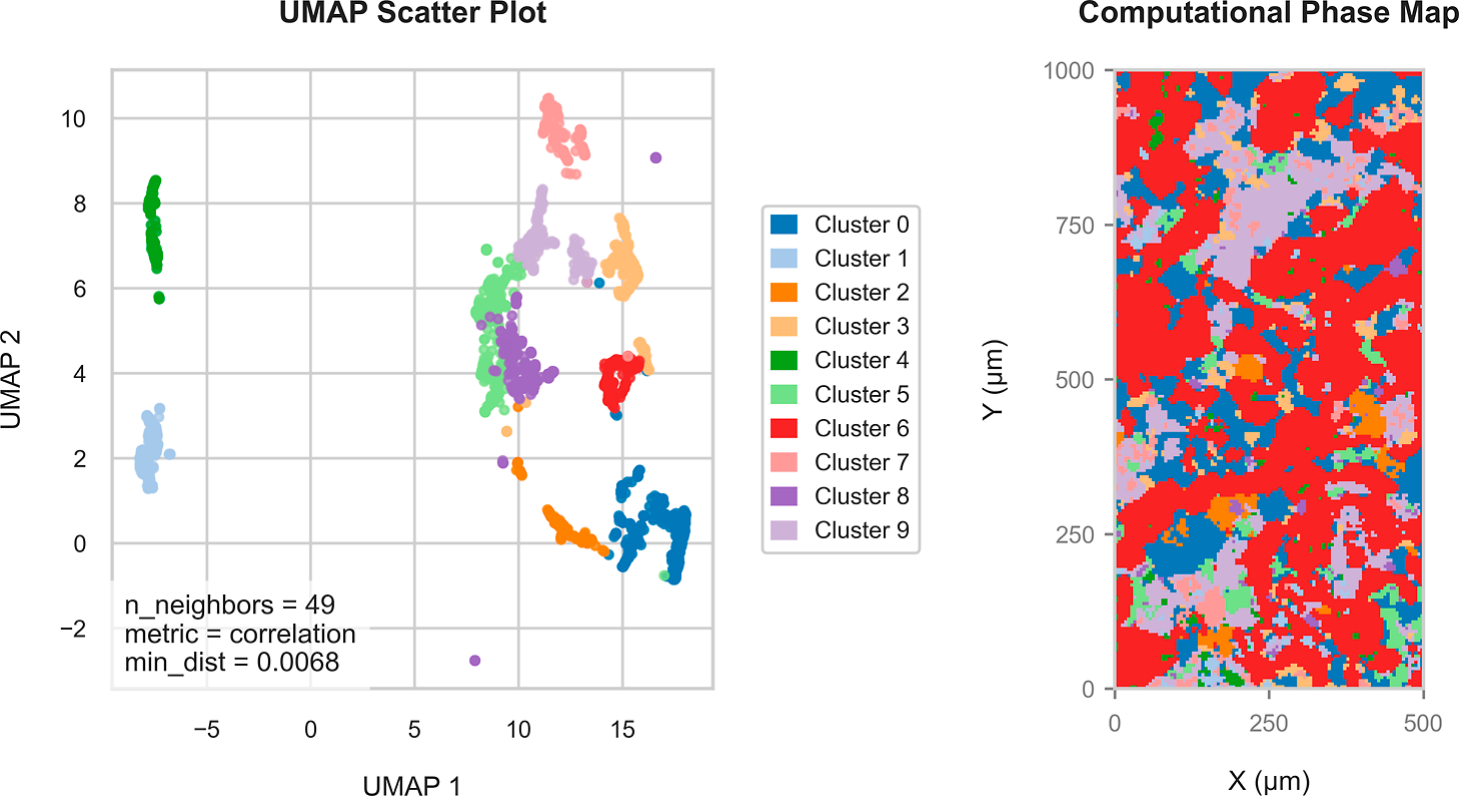
UMAP embedding and phase segmentation of the LA-ICP-TOFMS data set from “Layer 2” of the Poggio del Gatto sandstone. (Left) UMAP scatter plot of the high-dimensional data set, reduced to two dimensions using Uniform Manifold Approximation and Projection (UMAP) with the optimized hyperparameters. Subsequent *k*-means clustering partitions the data into 10 distinct groups. Each point represents an individual region, defined by Otsu thresholding, colored by its assigned cluster. (Right) Computational phase map showing the spatial distribution of the identified clusters across the sample area. The map uses the same color scheme as the UMAP plot to denote cluster identity.

Three aspects were critical to robust clustering: First, hyperparameters such as min_dist and n_neighbors demand careful tuning. However, the Optuna-based optimization framework provides the flexibility to probe broad parameter ranges and rapidly assess their impact. Second, extracting geologically meaningful regions at the outset proved essential, both for revealing interpretable patterns and for reducing computation time, because UMAP then operates on a smaller, more relevant data set. Third, incorporating the RSD of major components within clusters, in addition to the silhouette score, is crucial to ensuring that trace-element composition is not overweighted during optimization.

Even when not all clusters are visually distinct in the UMAP embedding, we will demonstrate below that *k*-means applied to the reduced dimensions still separate geologically meaningful phases.

### Mineral Matching Based on Stoichiometry

After the initial clusters were identified, they were matched to a set of predefined minerals using stoichiometric ratios. Match quality was evaluated by inspecting the differences in the cosine distance between each cluster and its candidate minerals. This procedure is designed to minimize reliance on geological intuition, enabling more straightforward phase identification. In principle, the workflow could be run “blindly”, comparing clusters against a large database of minerals’ stoichiometric ratios. However, because LA-ICP-TOFMS data lack the required concentrations of O, this strategy is ineffective as soon as the cation ratios become too similar. When mineral phases differ strongly in cationic composition, assigning clusters is generally unambiguous; when they vary primarily in anions, additional preanalysis characterization may be required. We followed the approach with a predetermined list and achieved reliable matches: calcite and quartz were identified confidently, whereas plagioclase, consistent with its solid-solution behavior, proved more ambiguous. Figure 3 visualizes these results by showing the differences of Relative match confidence between the identified mineral phases. Kaolinite and white mica were not identified.

**Figure 3:**
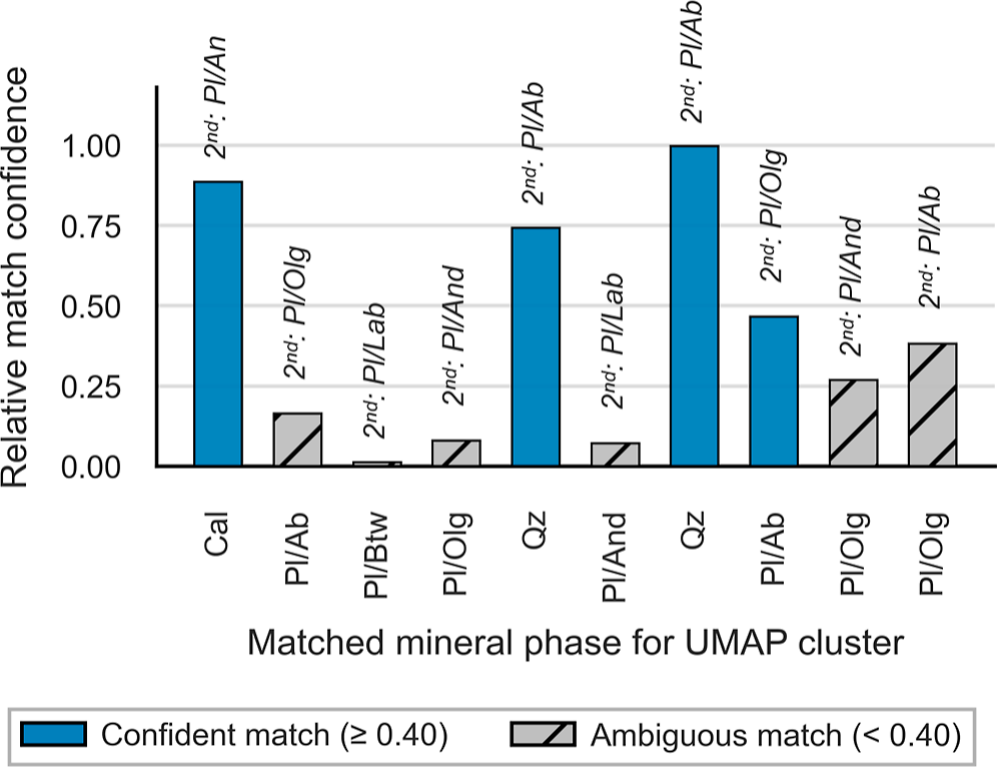
Visualization of Relative match confidence for cosine distances between the normalized stoichiometric ratios of the identified clusters shown here for the LA-ICP-TOFMS data set of “Layer 2” of the Poggio del Gatto sandstone. As to be expected, plagioclase end members (Pl/Ab, Pl/Olg, Pl/And) show ambiguity in comparison to calcite (Cal) and quartz (Qz).

The maps in Figure 4a,b illustrate a comparison between the petrographic image (i.e., reflected-light photomicrograph) and the computational phase map. Distinct features within the mineral are clearly visible in the phase maps and align with their petrographic counterparts. Beyond this visual correspondence, it is also essential to confirm the compositional consistency within each identified mineral phase. This is demonstrated through the RSDs of major and minor constituents within each phase, Figure 5, which include elements with a median concentration above 1 wt %. The data reveal that quartz is identified as a relatively pure phase in which Si RSDs are below 10%. In contrast, calcite exhibits compositional inhomogeneity, as shown by the variable mass fractions of Al and Si. However, primary component Ca remains below an RSD of 30%. Elevated RSDs observed in plagioclase endmembers are consistent with expected variable compositions. We stress that these RSDs incorporate measurement-related variability, which is inherent in any LA-ICP-MS analysis.

**Figure 4:**
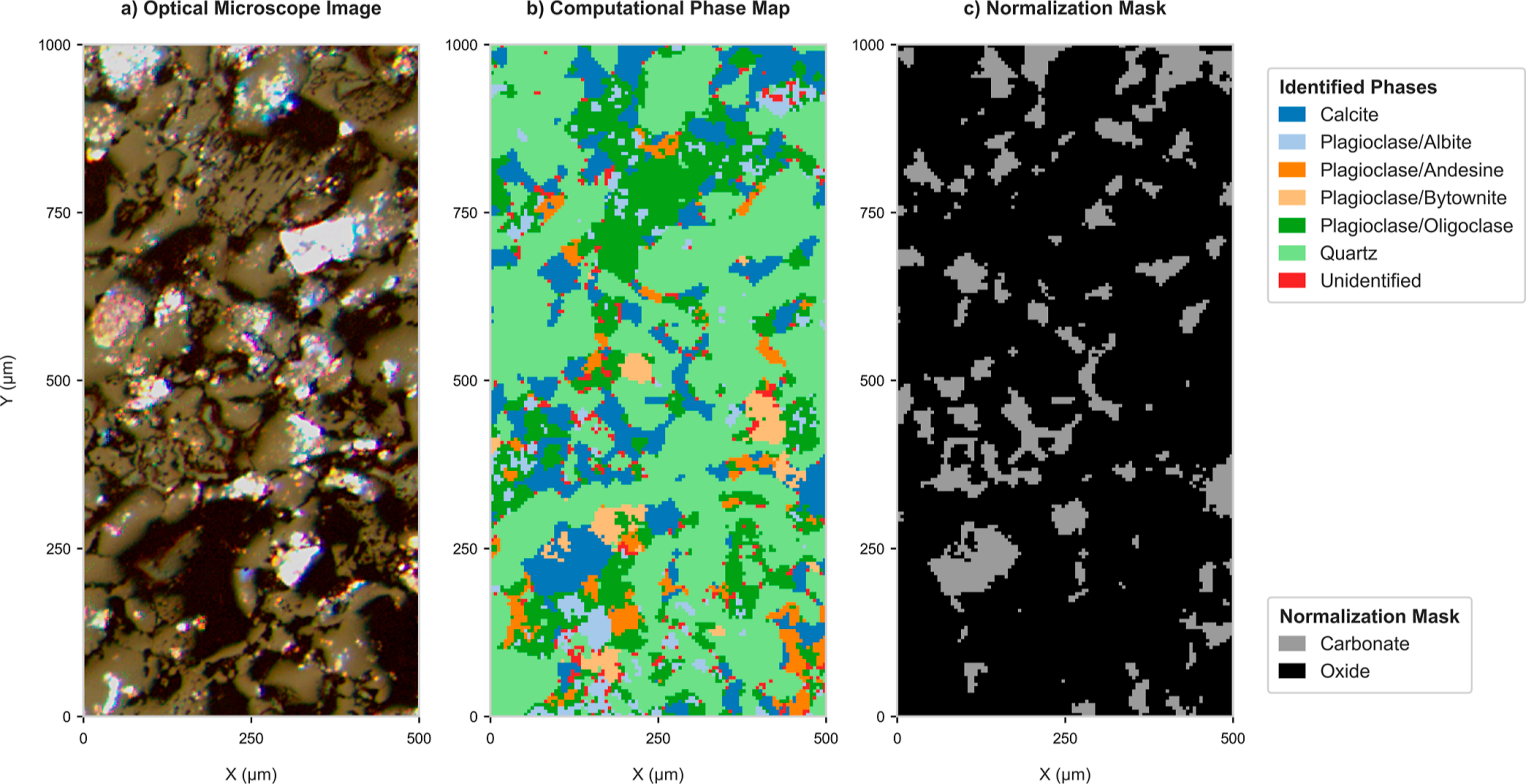
Validation of UMAP-derived mineral segmentation for the LA-ICP-TOFMS images of “Layer 2” of the Poggio del Gatto sandstone. Comparison between (a) the reflected-light photomicrograph, (b) the computational phase map constructed after merging the matched minerals, and (c) the normalization mask to be applied for carbonate-based 100 wt % normalization.

**Figure 5:**
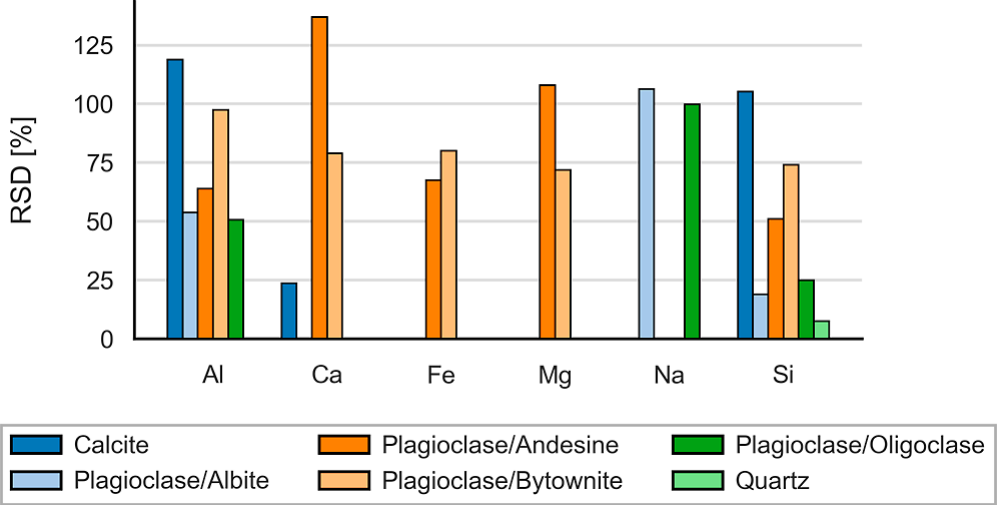
Relative standard deviations (RSDs) of major and minor elemental components for mineral phases identified in the “Layer 2” LA-ICP-TOFMS data set of the Poggio del Gatto sandstone. The bar chart summarizes intraphase compositional variability.

The normalization mask, which is generated based on the identified mineral phases, can be seen in Figure 4c. In the case of the investigated Poggio del Gatto sandstone, the only carbonate phase present is calcite.

### Renormalization Based on Carbonates

Applying an oxide-based normalization to carbonate samples will introduce composition-dependent biases that cannot immediately be generalized as a singular numerical value. The difference between the molecular masses of carbonate CO_3_^2–^ and its oxide equivalent O^2–^ yields an intrinsic bias on the order of a factor of about two, depending on the stoichiometry. Once this data is renormalized to a total of 100 wt %, the magnitude of the error becomes highly dependent on the specific elemental composition of the sample. An exemplary calculation illustrating this effect for a representative calcite pixel is provided in Table S2, yielding a relative error of 79%. In practice, this error varies across the image according to the local phase composition. Figure 6 illustrates the impact of normalization approaches for layer 2 of the samples as well as the spatial distribution of the resulting relative error. The figure presents Ca and Si elemental maps in two rows, while the first three columns compare different normalization strategies: normalization based on all oxides, normalization based on all carbonates, and a pixel-wise normalization guided by mineral phase identification, as outlined in the workflow proposed in this study. The final column displays the relative error between the bulk oxide- or carbonate-based normalization and the pixel-wise, phase-informed normalization.

**Figure 6:**
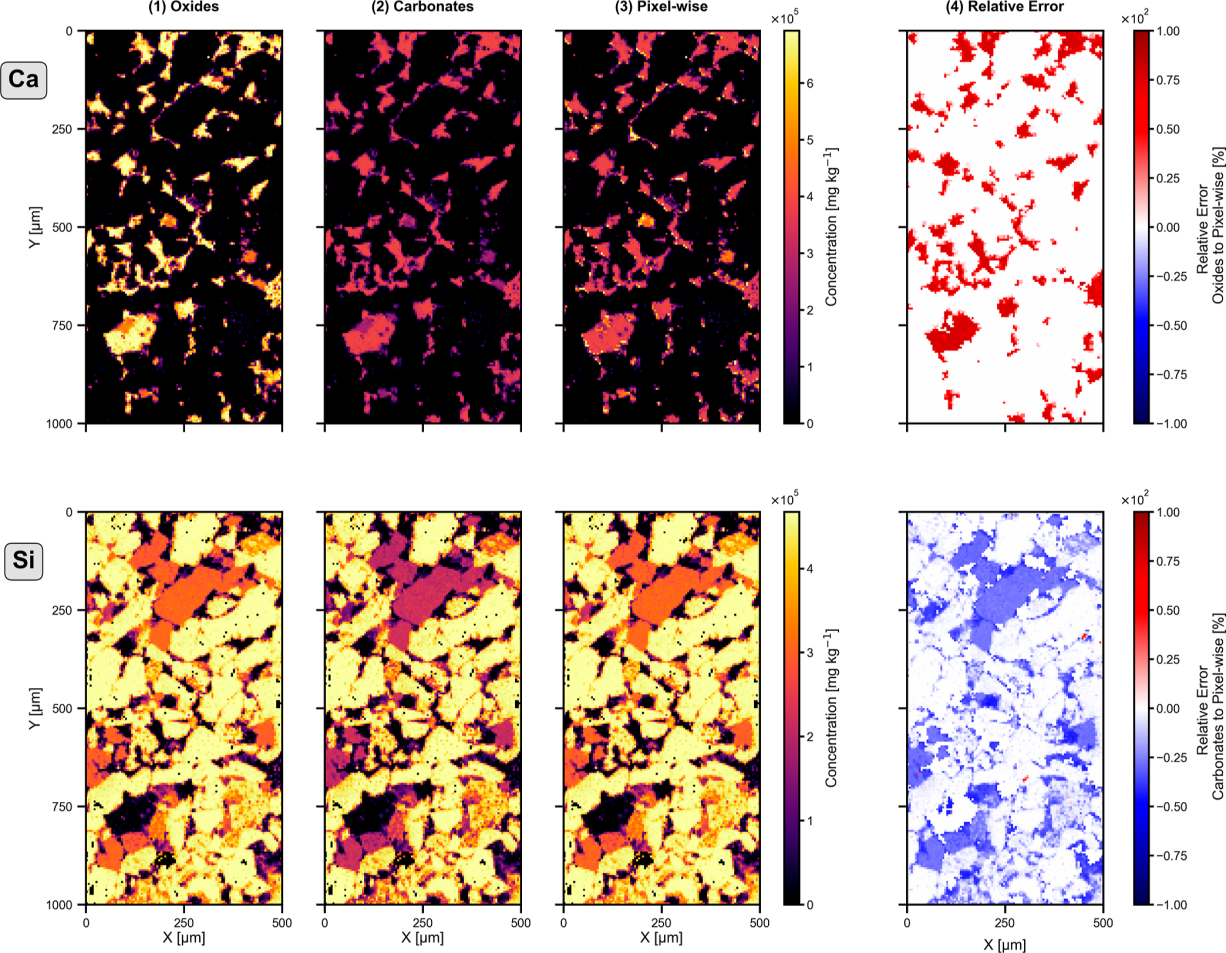
Comparison of normalization strategies for Ca and Si LA-ICP-TOFMS elemental maps in “Layer 2” of the Poggio del Gatto sandstone. Rows show Ca (top) and Si (bottom); columns compare (1) oxide-based normalization, (2) carbonate-based normalization, (3) pixel-wise, phase-informed normalization (this study), and (4) relative error between the first two and the phase-informed approach.

For Ca (in this matrix primarily associated with calcite, a dominant carbonate phase in our samples), the relative error depicted quantifies the effect of applying oxide-based normalization across the entire image. This results in a systematic overestimation of the Ca concentration by approximately 60%. Conversely, for Si—in this matrix found mainly in quartz and various plagioclase phases—the error is shown for applying carbonate-based normalization across an oxidic matrix. In such cases, the Si concentrations are underestimated by roughly 10% to 40%, depending on the mineral phase.

These findings highlight that using a single normalization scheme across heterogeneous samples introduces substantial quantification errors. To mitigate this, the pixel-wise phase segmentation enabled by the proposed UMAP-based workflow offers a significant advantage, especially in fine-grained matrices. In short, phase-aware, pixel-wise normalization combines the strengths of both normalization approaches and proves to be highly beneficial.

### Interpretability across Layers

In this case, each elemental image of each of the four thin sections (“Layer 1” to “Layer 4”) contains 20,000 pixels (100 × 200 pixels), each representing a multielement signal. Without reliable phase identification, this information cannot be used effectively for quantitative geological interpretation. Segmenting the image into mineral phases enables, in addition to the improved quantification accuracy, phasewise interpretation across samples. In the case of the Poggio del Gatto sandstone, this approach allows elemental trends to be visualized for the four layers and for each mineral phase. Figure 7 presents Al, Sr, and Mn median concentrations across “Layer 1” to “Layer 4” and normalized to “Layer 1”. These elements were selected for their geological relevance and analytical reliability in LA-ICP-MS. The plots highlight that the interpretation of element concentrations depends on whether bulk values (first column, no phase segmentation) or phase-resolved values are considered. For instance, in the bulk column, the mass fractions of Mn peaks at “Layer 3” are approximately three times those of “Layer 1” (Figure 7c). Phase segmentation shows that this compositional difference corresponds to a significant increase in plagioclase abundance and in a relatively minor calcite concentration. Similar phase-dependent differences are observed for Al and Sr, showing the value of phase-resolved analysis over bulk averages.

**Figure 7:**
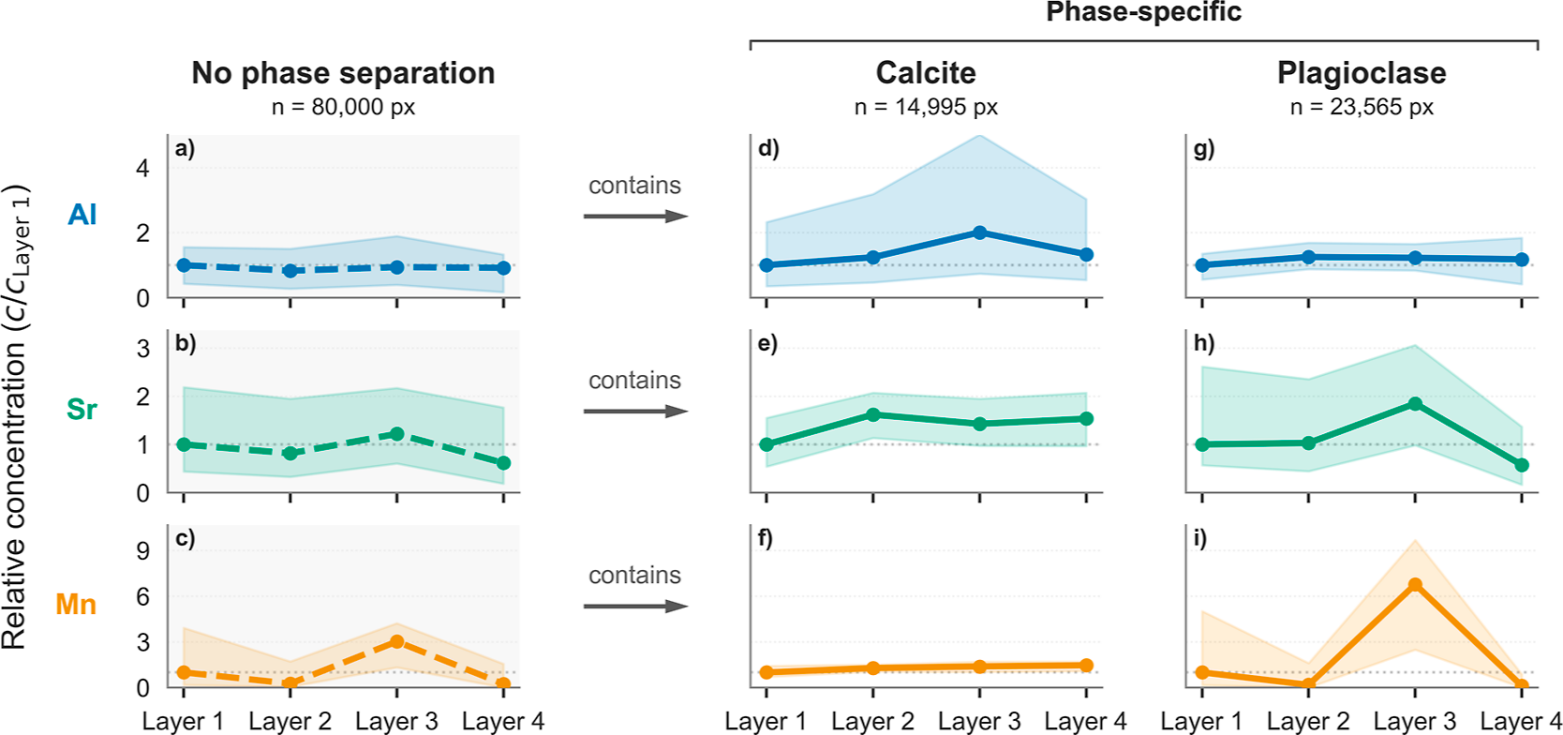
Elemental concentration trends across Layers 1–4 of the Poggio del Gatto sandstone, derived from LA-ICP-TOFMS data. (a–c) All pixels pooled (no phase segmentation). (d–f) Calcite-only pixels. (g–i) Plagioclase-only pixels, while all endmembers are combined. Values are relative elemental concentrations, normalized to the corresponding concentration in “Layer 1”. Calculations based on medians; error bars span the interquartile range (25th–75th percentiles). *n* denotes the number of pixels used per phase.

In summary, our analysis significantly improves the interpretation of mineralogical and chemical compositions of geological samples, especially those that experienced intense fluid–rock interactions. In the case of the Poggio del Gatto sandstone, the changes of mineral and chemical compositions have been captured at the scale of few cm using a large data set (e.g., ∼15,000 calcite pixels). These changes might be critical to constrain the effects of dawsonite precipitation in this rock.

## Conclusion

This study demonstrates that phase-specific normalization, guided by unsupervised phase segmentation of LA-ICP-MS images, can substantially improve the accuracy of elemental quantification in fine-grained geological matrices. Rather than relying on a single global assumption (e.g., all oxides or all carbonates), the workflow adapts normalization to the actual mineralogy, minimizing systematic errors and enabling phase-resolved geochemical interpretation.

The segmentation step, based on UMAP embedding and *k*-means clustering, is fully unsupervised. However, the subsequent matching of clusters to specific minerals still relies on a limited set of preidentified stoichiometries and is therefore not yet fully unsupervised. Expansion to larger stoichiometric databases could help overcome this limitation and move toward automated mineral classification. A key challenge remains the similarity of cation ratios in certain mineral groups, which may still require complementary data to ensure robust assignments.

Overall, beyond the specific case of the Poggio del Gatto sandstone presented in this study, this work provides a framework for quantitative elemental imaging of complex geological samples and is readily transferable to other contexts that require spatially resolved composition-aware normalization. This contribution adds a practical implementation to a growing literature that already uses multivariate statistics to unlock the immense information depth of LA-ICP-TOFMS.

## Supplementary Material



## Notes

Mineral stoichiometry, data processing optimization, elemental mapping, phase segmentation, and normalization calculations (PDF)
