# Supplementary material for: Mineral Phase-Resolved Quantification in LA-ICP-MS Imaging
Source: Anal Chem. 2025 Dec 17;98(1):581–9. doi: 10.1021/acs.analchem.5c05398 (PMC12809639; doi:10.1021/acs.analchem.5c05398)
Supplement: Supplementary file 1 [file ac5c05398_si_001.pdf]

Supporting Information

Mineral phase-resolved quantification in LA-ICP-MS imaging

Barbara Umfahrer[1], Jakub Buday[2], Pavel Pořízka[2], Jozef Kaiser[2], Paolo S. Garofalo[3]*, Detlef Günther[1]*

[1]Department of Chemistry and Applied Biosciences, Laboratory of Inorganic Chemistry, ETH Zurich, Vladimir-Prelog-Weg 1, 8093, Zurich, Switzerland
[2]Central European Institute of Technology (CEITEC) Brno University of Technology, Purkyňova 123, Brno 612 00, Czech Republic
[3]Università degli Studi di Bologna, Dipartimento di Scienze Biologiche, Geologiche ed Ambientali, Via Zamboni 67, 40126 Bologna, Italy

*Corresponding authors
guenther@inorg.chem.ethz.ch, paolo.garofalo@unibo.it

Table of contents

	**Description**	**Page**
**Figure S1**	*Poggio del gatto* sandstone	S2
**Table S1**	Stoichiometry of ideal mineral composition identified by XRD	S3
**Figure S2**	Optimization of UMAP	S3
**Figure S3**	LIBS elemental intensity maps "Layer 1"	S4
**Figure S4**	LIBS elemental intensity maps "Layer 2"	S5
**Figure S5**	LIBS elemental intensity maps "Layer 3"	S6
**Figure S6**	LIBS elemental intensity maps "Layer 4"	S7
**Figure S7**	Scatter plot/computational phase map "Layer 1"	S8
**Figure S8**	Scatter plot/computational phase map "Layer 3"	S8
**Figure S9**	Scatter plot/computational phase map "Layer 4"	S9
**Table S2**	Renormalization shown on one calcite pixel	S10

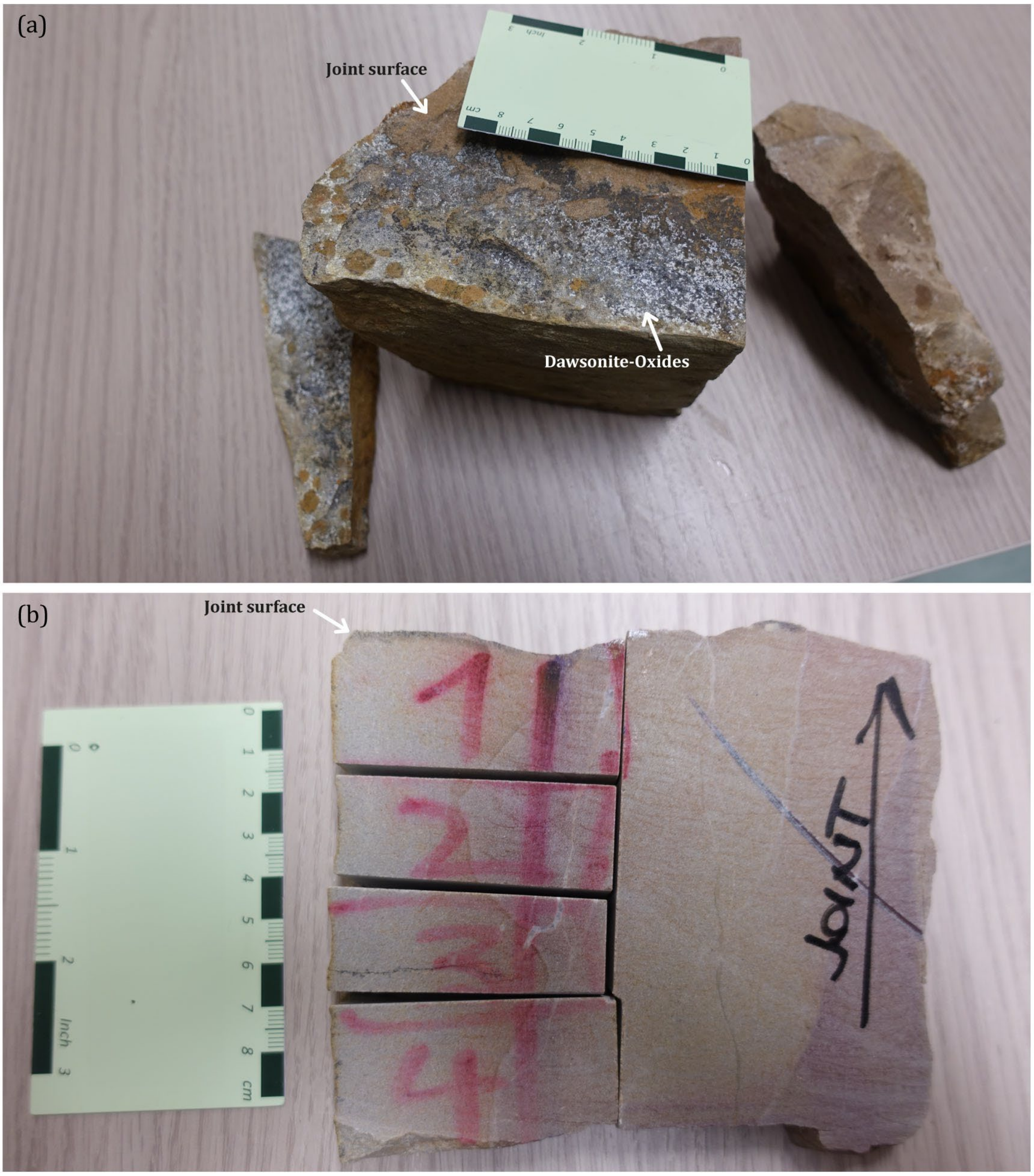

Figure S1. Poggio del Gatto *sandstone from Mt. Amiata, Italy. (a) Top view with Dawsonite and oxide phases. (b) Side view showing thin-section sampling positions “Layer 1” to “Layer 4” at increasing distance from the joint surface.*

Table S1. Stoichiometry of ideal mineral composition identified by XRD

**Mineral**	**Composition**
Quartz	SiO_2
Calcite	$CaCO_3$
Plagioclase	$(Na,Ca)[(Si,Al)AlSi_2]O_8$
Kaolinite	$Al_2(Si_2O_5)(OH)_4$
Mica/illite	$KAl_3Si_3O_{10}(OH)_2$

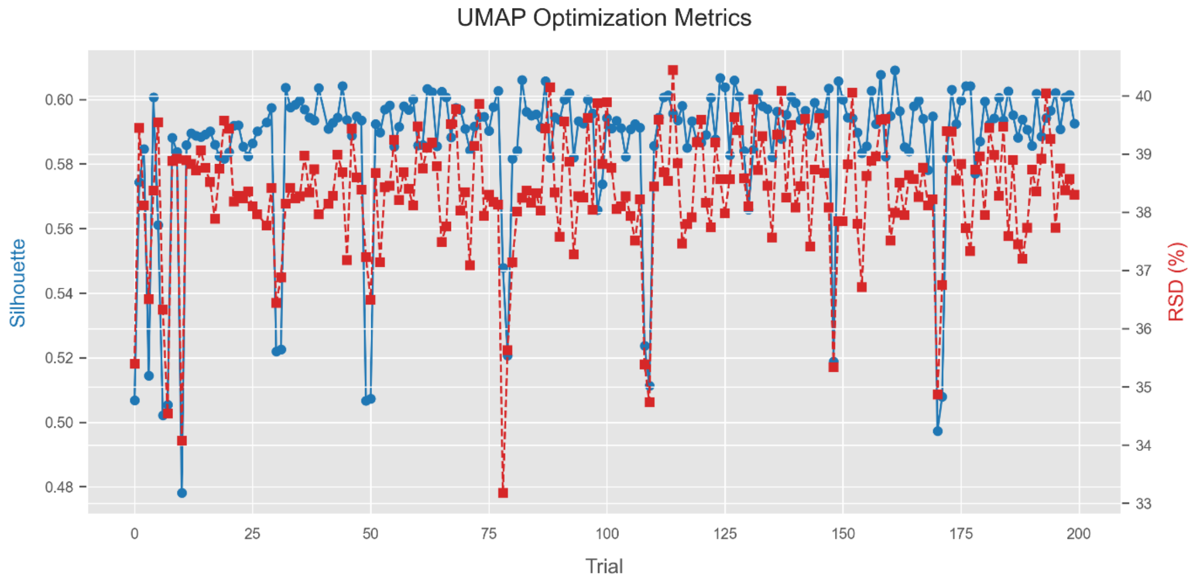

Figure S2. Optimization of UMAP and k-means based on the silhouette score and the Relative Standard Deviation (RSD) within an identified cluster. The optimization process converges at 20-30 trials.

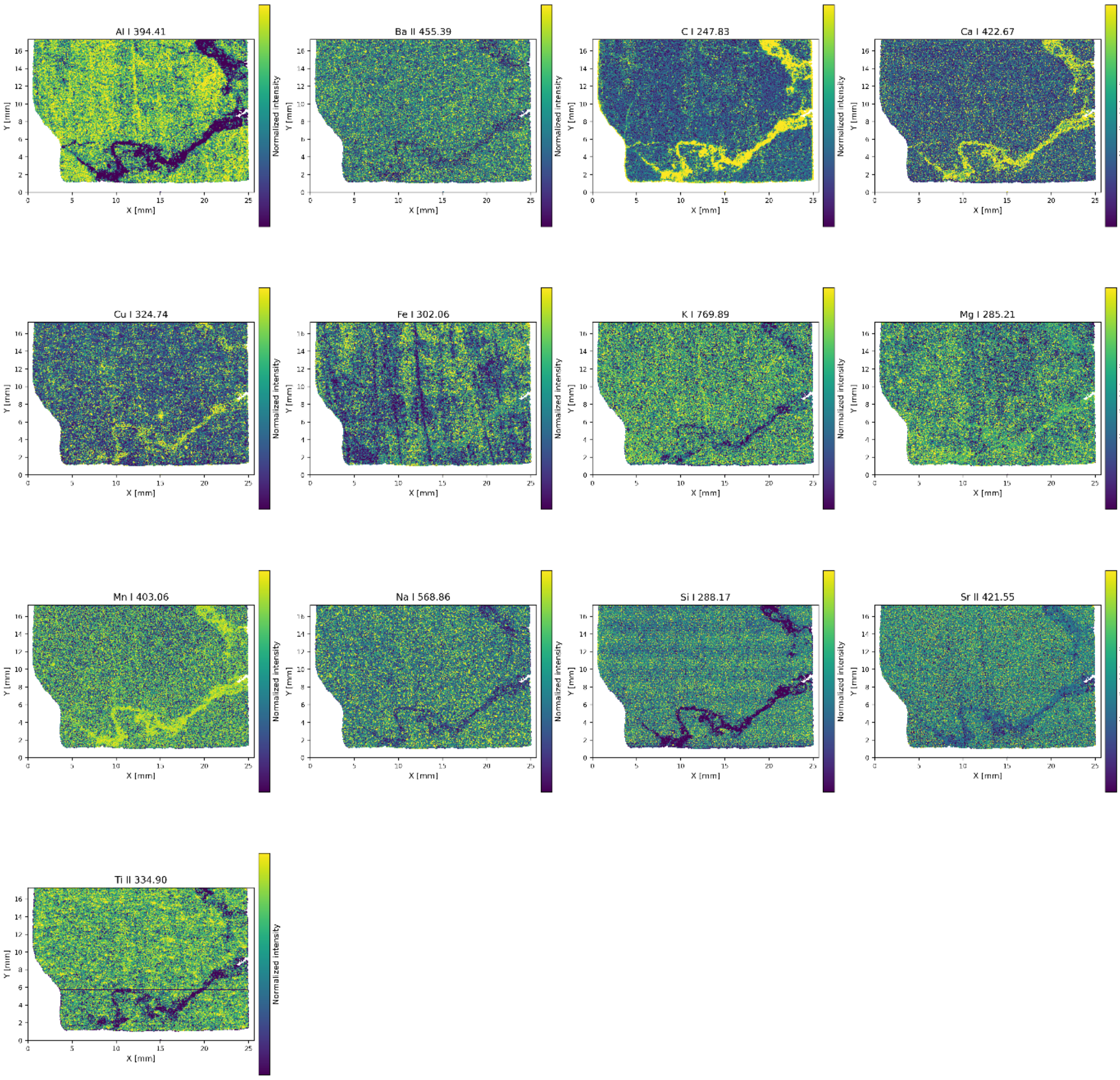

Figure S3. LIBS elemental intensity maps of thin section "Layer 1".

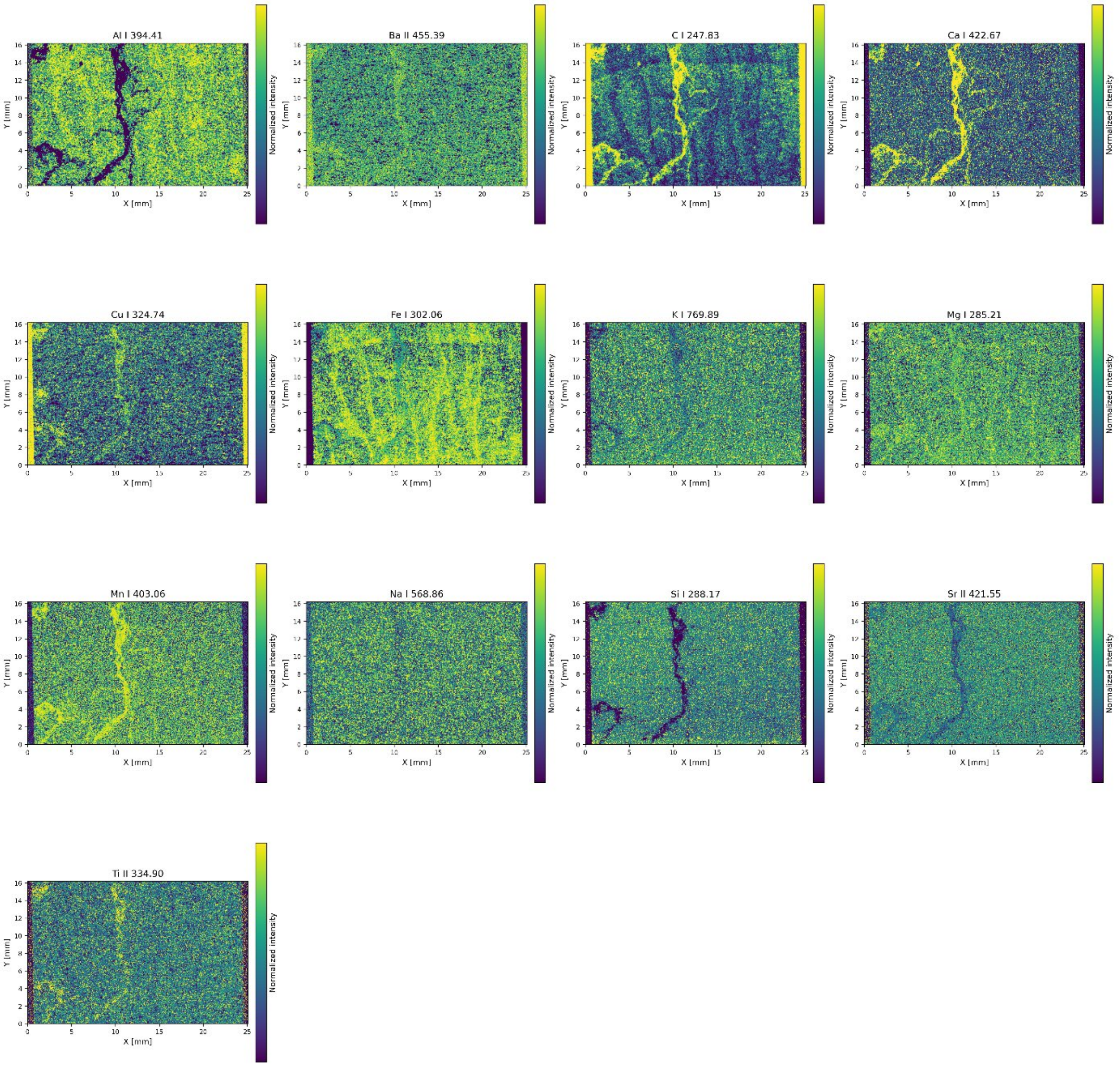

Figure S4. LIBS elemental intensity maps of thin section "Layer 2“ mounted on a microscope slide.

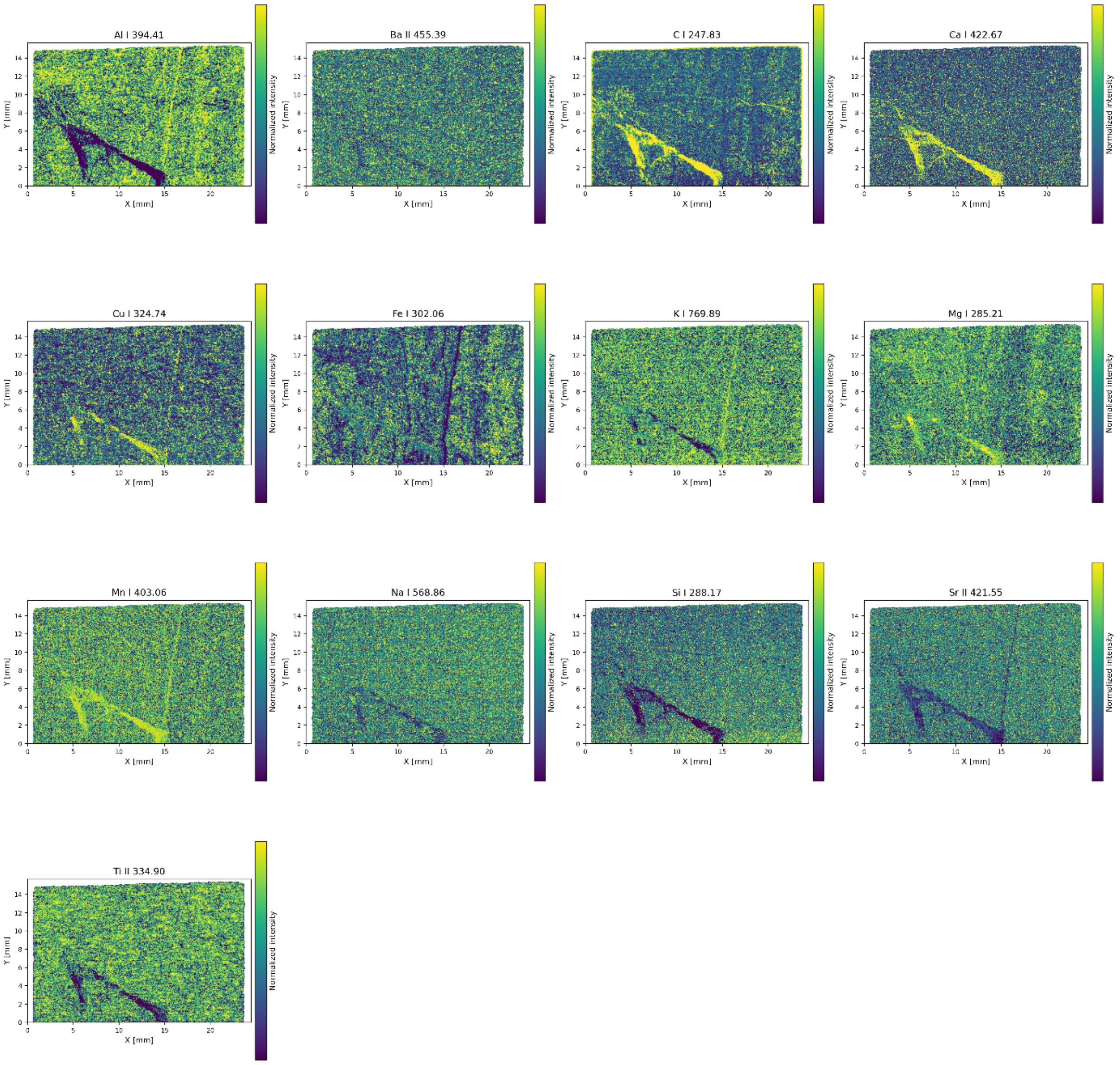

Figure S5. LIBS elemental intensity maps of thin section "Layer 3".

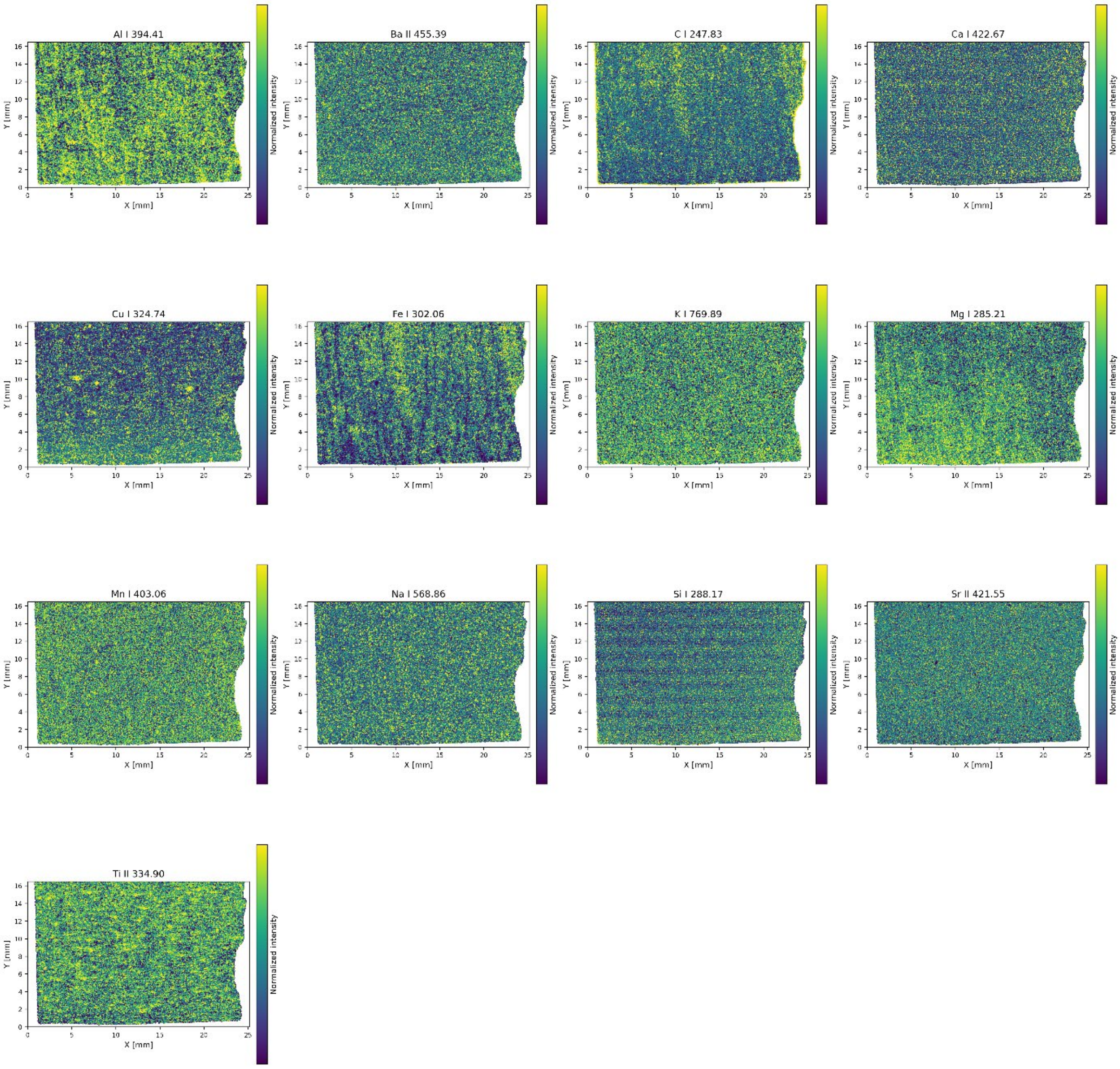

Figure S6. LIBS elemental intensity maps of thin section "Layer 4".

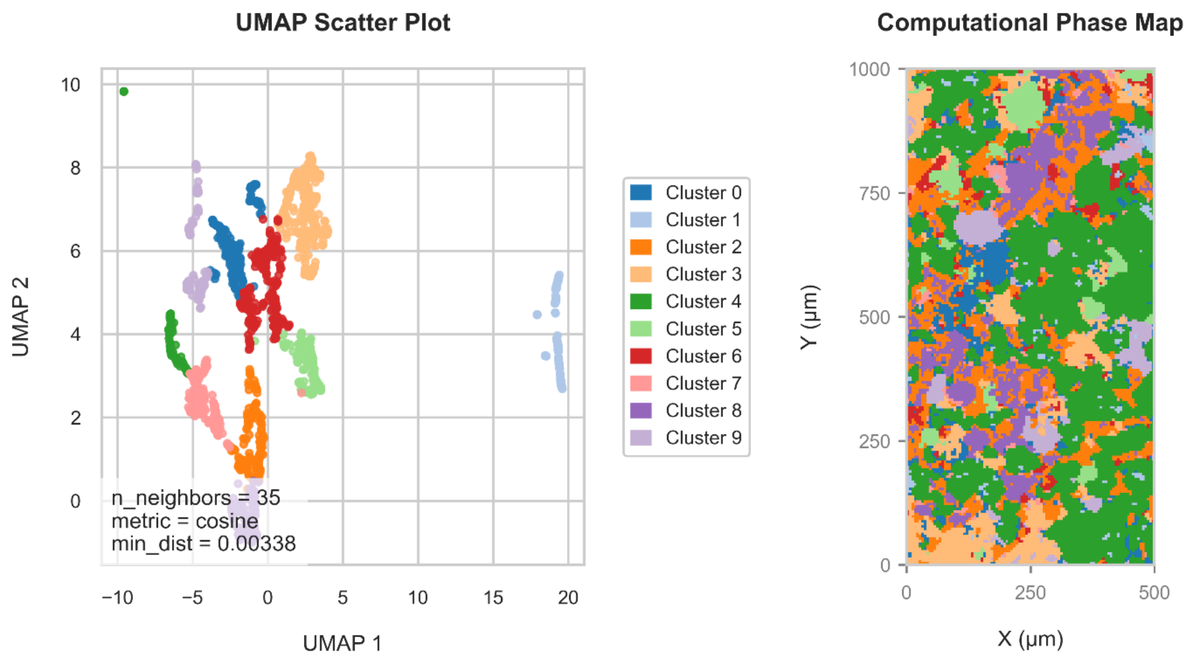

Figure S7. Scatter plot after optimized UMAP and generated computational phase map for "Layer 1".

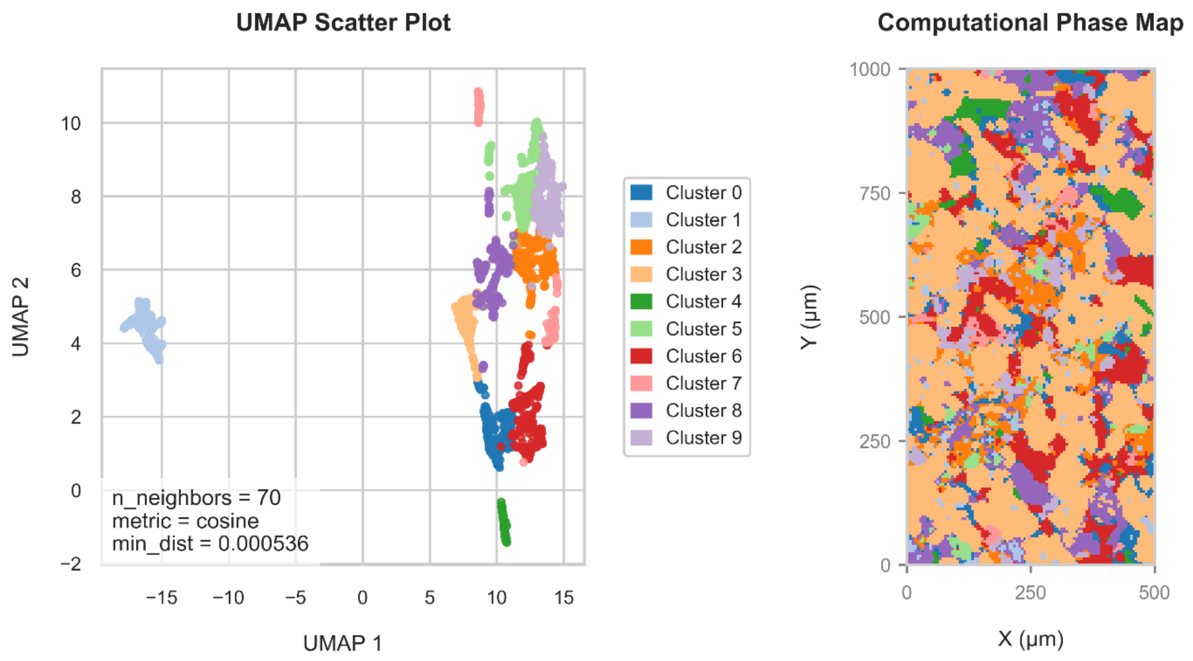

Figure S8. Scatter plot after optimized UMAP and generated computational phase map for "Layer 3".

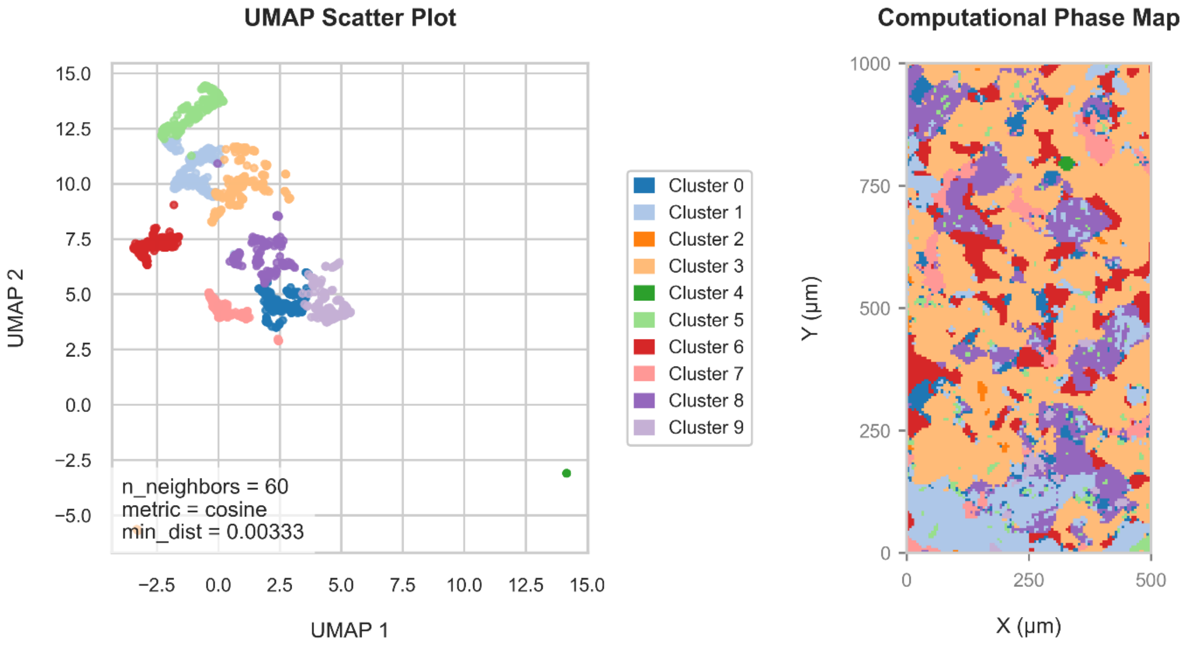

Figure S9. Scatter plot after optimized UMAP and generated computational phase map for "Layer 4".

To clarify the impact on quantification, we selected a random pixel of the calcite phase as an example and computed the deviation that arises when a carbonate data point is quantified as an oxide: the relative error. The proof of concept for the normalization approaches was presented previously (Leach and Hieftje (2000), Liu et al. (2008), Chen et al. (2011); see *Introduction*). For this specific application and sample, the scheme of calculation is reported in the following Table S2.

Table S2. Quantification deviation when a carbonate pixel is calculated as an oxide. Comparison of calculated element concentrations expressed as oxides and as mainly carbonates for one representative calcite pixel. Concentrations are normalized to 100 wt% oxides and 100 wt% carbonates, respectively. "Absolute deviation" refers to the difference between the element concentrations obtained under the two normalization schemes, and "Relative error" expresses this deviation as a percentage.

	**Concentration as oxide norm. to 100 wt% oxides [mg kg^{-1}]**		**Element concentration norm. to 100 wt% oxides [mg kg^{-1}]**		**Concentration as carbonate [mg kg^{-1}]**	**Concentration as carbonate norm. to 100 wt% carbonates [mg kg^{-1}]**		**Element concentration norm to 100 wt% carbonate [mg kg^{-1}]**	**Absolute deviation [mg kg^{-1}]** = Elem. conc. (oxide) – Elem. conc. (carb.)	**Relative error [%]** = (Absolute deviation / Elem. conc. (carb.)) * 100
CaO	968021	*Ca*	691235	*$CaCO_3$*	1726217	966430	*Ca*	386991	304244	79
SiO_2	2792	*Si*	1304	*SiO_2*	2790	1562	*Si*	730	574	79
MnO	6505	*Mn*	5034	*$MnCO_3$*	10532	5896	*Mn*	2818	2216	79
Al_2O_3	12246	*Al*	6476	*$Al_2(CO_3)_3$*	28081	15721	*Al*	3626	2850	79
FeO	5215	*Fe*	4050	*$FeCO_3$*	8402	4704	*Fe*	2268	1783	79
K_2O	18	*K*	15	*K_2O*	18	10	*K*	8	6	79
MgO	4109	*Mg*	2476	*$MgCO_3$*	8589	4809	*Mg*	1386	1090	79
BaO	0.5	*Ba*	0.4	*$BaCO_3$*	0.6	0.3	*Ba*	0.2	0.2	79
SrO	1074	*Sr*	907	*$SrCO_3$*	1528	856	*Sr*	508	399	79
Ho_2O_3	2.1	*Ho*	1.9	*Ho_2O_3*	2.1	1.2	*Ho*	1.0	0.8	79
ThO_2	1.9	*Th*	1.7	*ThO_2*	1.9	1.1	*Th*	0.9	0.7	79
PbO	15	*Pb*	14	*$PbCO_3$*	18	10	*Pb*	7.6	6	79
sum	**1000000**				1786179	**1000000**				
